# Pandemic prevention and unsustainable animal-based consumption

**DOI:** 10.2471/BLT.20.276238

**Published:** 2021-06-01

**Authors:** Harman S Sandhu, Anish Arora, Saadia I Sarker, Bindra Shah, Anusha Sivendra, Emily S Winsor, Anahat Luthra

**Affiliations:** a Institute of Health Policy, Management and Evaluation, University of Toronto, 155 College Street, Suite 425, Toronto, ON M5T 3M6, Canada.; b Faculty of Medicine, McGill University, Montréal, Canada.; c Toronto General Hospital, University Health Network, Toronto, Canada.; d Institute for Better Health, Trillium Health Partners, Mississauga, Canada.; e Milken Institute School of Public Health, The George Washington University, Washington DC, United States of America.; f Canadian Agency for Drugs and Technologies in Health, Ottawa, Canada.; g Schulich School of Medicine & Dentistry, Western University, London, Canada.

The health, economic and social crisis caused by the coronavirus disease 2019 (COVID-19) pandemic has disrupted lives globally and has had a particularly adverse impact on disadvantaged groups.^1^ A United Nations Environment Programme (UNEP) report published in July 2020 emphasized the importance of examining the initiating causes leading to the spread of COVID-19 as a way to prevent future pandemics.^1^ For example, the zoonotic origin of COVID-19 is hypothesized to have been a wet animal market in Wuhan City, China, where a large number of infected people were exposed during the early stages, with bats and birds being the potential intermediary link between animals and humans.^2^ Although the current public health response is focused on preventing human-to-human transmission, there is less discourse on how factors at zoonotic origins of the disease can be modified.^1^ The production, distribution and consumption of animal-based products (such as meat, dairy and eggs) is a major risk for zoonotic transmission.^1^ Therefore, our perspective is that promoting sustainability, particularly by reducing unsustainable animal-based consumption, must be part of the broader response to reduce the risk of future pandemics. We believe that our current path in relation to animal-based consumption is unsustainable, in that it may accelerate serious risks to human and environmental health. This perspective is drawn from the collaborative and multidisciplinary One Health approach, which focuses on improving the health of people, animals and environments.^1^ We present dietary patterns, discuss how these trends influence risk of zoonotic transmission and advocate for a reduction in unsustainable animal-based consumption to be included in future research and policy.

## Diet types and global patterns

Most of the world’s population follows an omnivorous diet, which consists of both animal- and plant-based food.^3^ Less than 10% of the global population follows a plant-based diet, that is either vegetarianism (which excludes meat) or veganism (complete exclusion of any animal-based product).^3^^,^^4^ Maintaining a plant-based diet over an omnivorous diet has nutritional benefits, such as an improved dietary health index and increased intake of a variety of vitamins and minerals.^3^ Plant-based diets are also associated with numerous long-term health benefits including lower levels of body mass index, as well as lower risk of ischaemic heart disease and cancer when compared to omnivorous diets.^4^ Spreading and scaling the production and uptake of nutritious plant-based foods may be a key factor in achieving the sustainable development goals (SDG) 2 and 12, which respectively aim to end hunger, achieve food security and improve nutrition, and promote sustainable consumption and production by 2030.^5^

Despite the potential benefits of plant-based diets, animal-based consumption has grown in recent decades, with the largest increases occurring in lower- and middle-income countries such as China and Brazil.^6^ Furthermore, differences in diets are driven by several intersecting factors such as demographics (including socioeconomic status, geographical location and food accessibility), cost of food and policies, such as taxes.^4^^,^^6^ Other factors such as culture, religion, individual preferences and attitudes can also influence diets.^6^ For example, the consumption of wildlife meat in Africa and Latin America can be a result of the belief that wild meat is fresh, natural, traditional and safe.^1^ Alternatively, the practice of vegetarianism can be influenced by religious beliefs (such as Hinduism and Jainism), moral concerns about the cruel treatment of animals, environmental sustainability and health benefits.^7^

## Infectious disease transmission

Global urbanization and growing population are well noted phenomena.^1^^,^^5^^,^^6^ Both can drive higher food consumption and changes in dietary behaviour towards more animal-based consumption.^6^ We hypothesize that these phenomena will increase activities such as farming, hunting, harvesting and trading of animal products where there is a risk of zoonotic transmission. Among new and emerging diseases affecting humans, an estimated 75% are suspected to have animal origins (such as the Middle East respiratory syndrome in bats and the Zika virus in primates).^1^ Animals can also be intermediary hosts, where they get diseases from other animals and transmit it to humans, sometimes without getting sick. This form of transmission is particularly concerning in our complex food-chains, where humans may not be coming into direct contact with original animal hosts but can still get infected.^1^ Risks exist in farms where animal products are being produced and in markets where they are being sold. The risk of human-to-human transmission is also heightened in places of animal product processing. In the United States of America, 16 233 cases of COVID-19 and 86 deaths among workers in 239 meat and poultry processing facilities were recorded as of May 2020.^8^ Public health strategies to protect these workers and preserve the critical meat and poultry infrastructure have been identified as a priority.^8^ However, the status quo of unsustainable animal-based consumption remains unchallenged.

## Links to other threats

Intensified farming to meet the demand for animal-based consumption could exacerbate other global health threats such as antimicrobial resistance and climate change. Pathogenic antimicrobial-resistant bacteria can be transmitted to humans via food-chains and through wastewater from farms, hospitals and pharmaceutical facilities.^9^ Rising population levels, livestock production and associated antibiotic use, as well as increasing animal-based consumption, pose serious economic and public health risks.^9^ Moreover, the leading contributor of climate change is greenhouse gas emissions. Omnivorous diets contribute twice the amount of greenhouse gas compared to those that do not include meat.^10^ Vegan diets produce the least amount of greenhouse gas while also potentially reducing overall high-risk contact with animals and their products.^10^

## Research and policy directions

The multisector, collaborative One Health approach is crucial in addressing the COVID-19 pandemic and preventing future outbreaks; however, incorporating such an approach into policy design and implementation remains a challenge and does not appear to have wide adoption. Recent reports from UNEP and the World Health Organization detail several promising interventions and directions for preventing future pandemics by moderating factors of zoonotic transmission.^1^^,^^11^ Key recommendations include implementing One Health in policies, improving biosecurity and safe handling practices in wildlife trade and animal markets, and increasing surveillance of high-risk pathogens in animal populations. These reports offer short-term, potentially effective measures, whose implementation would require collaboration between well resourced actors (governments, researchers, industries and nongovernmental organizations). However, such reports neglect to discuss long-term strategies focusing on upstream factors, the role individual consumers can play, and how these strategies can be achieved in a post COVID-19 economic recovery period contributing to achieving SDG 2 and SDG 12 by 2030.

We present four directions for future research and policy activities and support concurrent implementation where possible. First, we call for further research to study the complex association between animal-based consumption, zoonotic transmission and pandemics. This linkage remains largely unexplored and researchers could draw from other studies such as the one that quantified greenhouse gas emissions associated with different diet types.^10^ Research is also needed to inform our second direction, which is to develop evidence-based educational campaigns that engage individual consumers. Current and newly gained knowledge that shows the risk of zoonotic transmission associated with diet types, as well as the benefits of reducing unsustainable animal-based consumption and of increasing plant-based consumption, should be communicated with individual consumers. Social media-based education and leader role-modelling could also help engage individuals in understanding their role in these efforts. While calls for policies targeted towards larger well resourced entities have been made,^1^^,^^11^ the need remains to bring individual consumers into the conversation so that they are informed about the evidence and what actions they can take. Third, equity and context must be prioritized because an uninformed reduction of animal-based consumption could cause food insecurity for those relying on it for their nutritional needs.^1^ In these cases, we urge actors such as governments, nongovernmental organizations, researchers and industry to collaborate and incentivize a reduction of animal-based consumption and investment into plant-based food where possible. Fourth, restructuring agricultural practices and food-chains may be a viable direction in a post-COVID-19 economic recovery period. As one third of croplands worldwide are used for animal feeds,^1^ repurposing them to produce food for human consumption may help meet the increasing population and demand for food as we recuperate from COVID-19.

Research and policy activities targeted to hotspot areas for zoonotic transmission could be most impactful. For example, East Asia, Latin America, South Asia, South-East Asia and sub-Saharan Africa are areas at risk for emerging pandemics, and also where animal-based consumption is increasing the most – compared with other regions and over time.^1^^,^^6^^,^^12^ Additionally, the development and implementation of any policies related to the reduction of unsustainable animal-based consumption will likely be complicated. Thus, those implementing such policies must assess and document the costs, benefits, acceptability and scalability of such reduction to build a stronger evidence base.^1^

To conclude, multiple actors need to critically examine the food-chain and related zoonotic transmission risks, while incorporating efforts to reduce unsustainable animal production, distribution and consumption. A One Health approach could bring many policy actors together to implement preventive measures upstream to promote sustainable animal-based consumption and reduce overconsumption. Plant-based diets are a promising tool for diverting investment away from unsustainable animal-based consumption and aiding in our response to global health threats such as pandemics, climate change and antimicrobial resistance.
